# Ectopie thyroïdienne hyoïdienne: à propos d’un cas chez une jeune fille malgache

**DOI:** 10.11604/pamj.2018.30.54.13523

**Published:** 2018-05-24

**Authors:** Sitraka Angelo Raharinavalona, Iandry Michella Razafinjatovo, Rija Eric Raherison, Thierry Razanamparany, Andriamparany Rasata Ravelo, Andrinirina Dave Patrick Rakotomalala

**Affiliations:** 1 Service d’Endocrinologie, Centre Hospitalier Universitaire Joseph Raseta de Befelatanana, Antananarivo, Madagascar; 2 Service de Médecine Nucléaire, Centre Hospitalier Universitaire Andohatapenaka, Antananarivo Madagascar; 3 Service d’Endocrinologie et de Cardiologie, Centre Hospitalier Universitaire Mahavoky Atsimo, Mahajanga, Madagascar

**Keywords:** Ectopie thyroïdienne, hyoïdienne, hypothyroïdie infraclinique, scintigraphie, Thyroid ectopia, hyoid region, subclinical hypothyroidism, scintigraphy

## Abstract

L'ectopie thyroïdienne est une pathologie rare liée à une défaillance de migration de la glande thyroïde lors de son développement embryonnaire. Elle est le plus souvent asymptomatique. Nous rapportons un cas d'ectopie thyroïdienne à localisation hyoïdienne chez une jeune fille malgache, révélée par une gêne esthétique, associée à une hypothyroïdie infraclinique. Le diagnostic était confirmé par l'échographie cervicale et la scintigraphie au technétium 99m. Même si l'hypothyroïdie est infraclinique, une hormonothérapie substitutive est de règle permettant ainsi d'obtenir une euthyroïdie biologique et de maintenir la bonne évolution clinique.

## Abstract (en)

Thyroid ectopia is rare. It results from a failure of migration of the thyroid gland during its embryonic development. It is most often asymptomatic. We here report a case of thyroid ectopia in the hyoid region in a young Malagasy girl, presenting as aesthetic discomfort associated with subclinical hypothyroidism. Diagnosis was confirmed using cervical ultrasound and scintigraphy with technetium-99m. Hormone replacement therapy is the gold standard even in patients with subclinical hypothyroidism because it results in physiologic euthyroidism and good clinical evolution.

## Introduction

L'ectopie thyroïdienne est une masse de tissu thyroïdien située en dehors de la loge thyroïdienne habituelle, le long du canal thyréoglosse [1]. Il s'agit d'une pathologie rare. Sa prévalence est estimée à 1 pour 100 000 à 300 000 patients hypothyroïdiens [2, 3]. Ainsi nous rapportons un cas d'ectopie thyroïdienne vu service de Médecine Nucléaire du CHU d'Andohatapenaka, Antananarivo, Madagascar.

## Patient et observation

**Figure 1:**
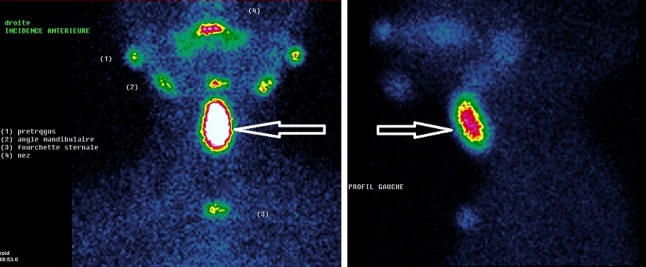
Photographie du cou de la patiente montrant la tuméfaction en position hyoïdienne (à gauche: cou en position normale et à droit: cou en extension)

**Figure 2:**
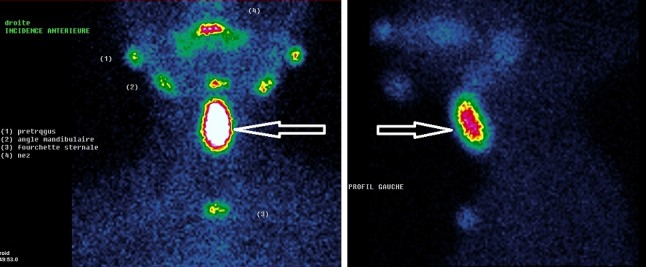
Scintigraphie au technétium 99m montrant l’absence totale de captation du radio traceur en regard de la loge thyroïdienne et la présence d’une fixation de façon homogène du traceur au-dessus de cartilage cricoïde (à gauche: vue de face et à droit: vue de profil)

Il s'agit d'une patiente âgée de 11 ans ayant consultée en Janvier 2013 pour une tuméfaction cervicale antérieure découverte par l'entourage, sans autre signe accompagnateur. Il n'y avait pas de trouble de croissance staturo-pondérale, ni psychomoteur. On ne notait dans ses antécédents aucune notion d'irradiation pendant l'enfance, ni de goitre familial. La patiente ne présentait pas de signes cliniques de dysthyroïdie notamment d'hypothyroïdie, ni de signes de compression tels une dyspnée, une dysphonie, une dysphagie ou de syndrome cave supérieur. A l'examen clinique, son poids était de 36kg pour une taille de 143 cm, soit un indice de masse corporelle de 17,6 kg/m^2^. La loge thyroïdienne était libre, mais on note la présence d'une masse molle, mobile à la déglutition, indolore, sans souffle vasculaire, située au niveau de la région hyoïdienne du cou donnant un aspect de « pomme d'Adam » (Figure 1). Il n'y avait pas d'autre malformation cliniquement décelable. A l'examen paraclinique on observe: 1) le bilan hormonal avait objectivé une hypothyroïdie infraclinique avec TSH à 8,1 µUI/mL (0,4 à 7) et T4 L à 9,3 pmol/L (4,8 à 12); 2) les autres examens biologiques standards étaient sans particularité (hémogramme, ionogramme sanguin, CRP, VSH, bilan lipidique); 3) l'échographie cervicale avait montré une vacuité de la loge thyroïdienne, un lobe gauche thyroïdien homogène d'un volume de 6,3cm^3^, en position haute paratrachéale. Le lobe droit était mal individualisé; 4) la scintigraphie au technétium 99m (Tc-99m) a mis en évidence l'absence de captation du radio-traceur en regard de la loge thyroïdienne et la présence d'une fixation homogène du Tc-99m, située au-dessus de cartilage cricoïde, correspondant à un parenchyme thyroïdien (Figure 2). Le taux de captation à la 20^e^ minute était de 1,5%.

Au total il s'agit d'une ectopie thyroïdienne hyoïdienne associée à une hypothyroïdie fruste ou infraclinique chez jeune fille de 11 ans révélée par une gêne esthétique. En raison de l'absence d'une indication chirurgicale, la patiente a reçu un traitement hormonal substitutif à base de L-thyroxine à la dose de 50μg/j, permettant ainsi d'obtenir une euthyroïdie biologique et de garder ses bons développements staturo-pondéraux et psychomoteurs.

## Discussion

Après la deuxième semaine de gestation, la glande thyroïde apparaît sous forme d'une prolifération épithéliale du revêtement endodermique au niveau de la partie médiane du plancher du pharynx primitif devenant par la suite le « foramen cæcum ». Elle suit progressivement une direction caudale et ventrale, le long du tractus thyréoglosse. A la septième semaine, elle atteint sa situation définitive au-dessous du cartilage thyroïdien, en regard des 5e et 6e anneaux trachéaux. Toute défaillance de cette migration peut mener à une ectopie thyroïdienne, dont la pathogénie reste encore mal élucidée [4, 5]. Les localisations ectopiques peuvent être sous mandibulaire, trachéale, cervicale latérale, carotidienne, œsophagienne, gastrique, duodénale, pancréatique, mésentérique, intracardiaque, aortique, pulmonaire, pituitaire, axillaire, et au niveau de l'iris de l'œil. Mais l'ectopie thyroïdienne sublinguale reste la forme la plus fréquente, retrouvée dans 70 à 90% des cas [1, 6]. Particulièrement à Madagascar, Randrianambinina et al ont déjà rapporté un cas de goitre ectopique médiastinal simulant un thymome, associé à une glande thyroïde cervicale normale [7]. Pour notre cas, il s'agit d'une ectopie thyroïdienne située au niveau de la région hyoïdienne. Il s'agit d'une affection rare qui touche surtout les femmes que les hommes avec un sex-ratio de 1/4 [2,8]. Les symptômes cliniques dépendent du volume et de la location de l'ectopie thyroïdienne et peuvent être associé ou non à une hypothyroïdie. Les manifestations le plus souvent observé au cours d'une ectopie thyroïdienne linguale sont à titre d'exemple la toux, la douleur, la dysphagie, la dysphonie, la dyspnée et l'hémorragie. Le retard statural et un des premiers signes d'hypothyroïdie chez le grand enfant. Ainsi, la réalisation d'un bilan thyroïdien doit se faire devant tout ralentissement de la croissance [1, 9]. Dans la majorité de cas, elle est asymptomatique [10], comme la nôtre.

Les imageries médicales permettent à la fois de confirmer le diagnostic d'une ectopie thyroïdienne, de planifier la stratégie thérapeutique et de faire le suivi du patient [1-3]. L'échographie cervicale met en évidence la vacuité de la loge thyroïdienne et éventuellement la présence d'une masse ectopique ayant la même échostructure que le tissu thyroïdien. La scintigraphie au technétium 99m ou mieux à l'iode 124 permet de mettre en évidence une hyperfixation en dehors de la loge thyroïdienne normale. Cette dernière reste la méthode de choix en termes de sensibilité et de spécificité [1,10,11]. Ces deux méthodes nous ont permis de confirmer le diagnostic et de déterminer la localisation de l'ectopie thyroïdienne de notre cas. Vu la rareté de cette entité clinique, il n'y a pas encore de consensus bien établi sur la stratégie de sa prise en charge [12]. Son traitement dépend de sa présentation clinique. En effet, l'ablation chirurgicale est indiquée en cas d'hémorragies graves itératives, de signes compressifs (dysphagie, dyspnée, dysphonie) et de suspicion de transformation maligne. Et l'hormonothérapie substitutive que nous avons opté pour notre patiente, est de règle en cas d'hypothyroïdie qui peut être infraclinique ou franche [5, 8]. Sur le plan évolutif, une dégénérescence maligne d'une ectopie thyroïdienne située au niveau des régions cervicales est toujours possible, même si le risque est faible [13]. Tout cela implique ainsi l'importance d'une surveillance clinico-biologique à long terme et d'une éventuelle cytoponction du tissu thyroïdien ectopique.

## Conclusion

L'ectopie thyroïdienne est une pathologie rare liée à une défaillance de migration de la glande thyroïde lors de son développement embryonnaire. Elle est le plus souvent asymptomatique d'où la nécessité d'une exploration radiologique qui est surtout baser sur la scintigraphie. Une hypothyroïdie infraclinique est une indication d'une hormonothérapie substitutive. La résection chirurgicale est réservée pour les formes compliquées.

## Conflits d’intérêts

Les auteurs ne déclarent aucun conflit d'intérêts.

## References

[1] Ghfir I, Guerrouj H, M'hamedi F, Ouboukdir R, Mouaden A, Ben Raïs Aouad N (2013). Double ectopie thyroïdienne explorée par imagerie scintigraphique en mode hybride TEMP/TDM : à propos d'un cas. Méd Nucl.

[2] Di Benedetto V (1997). Ectopic thyroid gland in the submandibular region simulating a thyroglossal duct cyst: a case report. J Pediatr Surg.

[3] Babazade F, Mortazavi H, Jalalian H, Shahvali E (2009). Thyroid tissue as a submandibular mass: a case report. J Oral Sci.

[4] Léger J (2007). Embryologie de la thyroïde et implication physiopathologique, In Chanson P, Young J dir. Traité d'endocrinologie.

[5] El Mazouni Z, El Wadeh I, Gaouzi A (2011). Ectopie thyroïdienne chez l'enfant. J Pédiatr puéricult.

[6] Guerra G, Cinelli M, Mesolella M, Tafuri D, Rocca A, Amato B (2014). Morphological, diagnostic and surgical features of ectopic thyroid gland: a review of literature. Int J Surg.

[7] Randrianambinina F, Rakotorahalahy R, Randrianambinina H, Ranaivomanana VF, Ramiandrasoa AL, Rakotoarisoa AJC (2015). Un goitre ectopique dans le mediastin antérieur avec un syndrome myasthénique simulant un thymome. J Fran Viet Pneu.

[8] Cherif L, Lakhoua Y, Khiari K, Hadj-Ali I, Rajhi H, Kaffel N (2004). L'ectopie thyroïdienne : à propos de deux cas. Ann Endocrinol.

[9] Gotlib J, Ianessi A, Santini J, Dassonvill O (2016). Ectopic lingual thyroid. Eur Ann Otorhinolaryngol Head and Neck diseases.

[10] Ibrahim NA, Fadeyibi IO (2011). Ectopic thyroid: etiology, pathology and management. Hormones.

[11] Konde SR, Singh BH, Pawar A, Sasane A (2012). Triple ectopic thyroid. MJAFI.

[12] Noussios G, Anagnostis P, Goulis DG, Lappas D, Natsis K (2011). Ectopic thyroid tissue: anatomical, clinical, and surgical implications of a rare entity. Eur J Endocrinol.

[13] Oguz A, Tuzun D, Ozdemir E, Ersoy R, Yazgan AK, Cakir B (2015). Importance of ectopic thyroid tissue detected in the midline of the neck: single center experience. Arch Endocrinol Metab.

